# Normalized Cut Group Clustering of Resting-State fMRI Data

**DOI:** 10.1371/journal.pone.0002001

**Published:** 2008-04-23

**Authors:** Martijn van den Heuvel, Rene Mandl, Hilleke Hulshoff Pol

**Affiliations:** Rudolf Magnus Institute of Neuroscience, Department of Psychiatry, University Medical Center Utrecht, Utrecht, The Netherlands; Freie Universitaet Berlin, Germany

## Abstract

**Background:**

Functional brain imaging studies have indicated that distinct anatomical brain regions can show coherent spontaneous neuronal activity during rest. Regions that show such correlated behavior are said to form resting-state networks (RSNs). RSNs have been investigated using seed-dependent functional connectivity maps and by using a number of model-free methods. However, examining RSNs across a group of subjects is still a complex task and often involves human input in selecting meaningful networks.

**Methodology/Principal Findings:**

We report on a voxel based model-free normalized cut graph clustering approach with whole brain coverage for group analysis of resting-state data, in which the number of RSNs is computed as an optimal clustering fit of the data. Inter-voxel correlations of time-series are grouped at the individual level and the consistency of the resulting networks across subjects is clustered at the group level, defining the group RSNs. We scanned a group of 26 subjects at rest with a fast BOLD sensitive fMRI scanning protocol on a 3 Tesla MR scanner.

**Conclusions/Significance:**

An optimal group clustering fit revealed 7 RSNs. The 7 RSNs included motor/visual, auditory and attention networks and the frequently reported default mode network. The found RSNs showed large overlap with recently reported resting-state results and support the idea of the formation of spatially distinct RSNs during rest in the human brain.

## Introduction

Functional brain imaging studies have suggested that the brain is not inactive during rest, but rather shows a default state of activation [1]–[5]. Low frequency oscillations (ranging from 0.01 to 0.1 Hz) of resting-state functional Magnetic Resonance Imaging (fMRI) time-series are known to show correlated patterns between anatomical separated brain regions [1], [6], [7]. These correlations are suggested to originate from coherency in the underlying neuronal activation patterns of these regions and believed to reflect functional connectivity. Regions that show this kind of coherent functional behavior are said to form a resting-state network (RSN). Multiple RSNs have been reported, including primary auditory, motor and sensory networks, attention networks and the default mode network [2], [5], [8], [9]. Resting-state fMRI patterns are traditionally examined by correlating the rest recorded fMRI time-series of a single seed voxel against the time-series of all other voxels, resulting in a functional connectivity map (fcMap). A seed voxel is usually selected from an activation map of a separately acquired fMRI experiment. For example, when the seed voxel is based on activation in a motor task, the resting-state fcMap gives information about functionally connected regions involved in the motor network. Several studies mapping motor, visual, auditory and even cognitive networks have shown the potential of this seed-based resting-state analysis [1], [6], [10], [11]. However, the information of a fcMap is limited to the network associated with the selected seed voxel. In contrast, model-free methods enable the exploration of spatial and temporal activation patterns without the need of defining a specific model. Several model-free methods have been applied to individual (resting) PET and fMRI data, including principal component analysis (PCA) [12], independent component analysis (ICA) ) [2], [13], [14], hierarchical [15] and Laplacian based clustering [16].

A few model-free resting-state group methods have been introduced [17]–[19]. However, group analysis of resting-state data is still a complex task and often involves human input in selecting the number of meaningful group networks. Here, we report on a model-free group graph clustering approach for selecting consistent functionally connected RSNs across a group of subjects. Our method works at the voxel level with whole brain coverage and includes a procedure to determine the number of RSNs as an optimal fit of the data. The method involves the formation of individual and group functional connectivity graphs which are clustered to group voxels that show high functional connectivity in RSNs. For the clustering itself, the normalized cut (Ncut) graph clustering method of Shi and Malik [20] was used. The Ncut criterion is an unbiased measure of the disassociation between the subgroups of the data and minimizing this criterion directly leads to maximizing the total association within the subgroups. The Ncut method has the strong advantage of being less sensitive to outliers than other graph clustering methods. In essence, our RSN group selection method consists of a two stage process, combining clustering at the individual level with clustering at the group level. At the individual level, the inter-voxel functional connectivity of an individual fMRI dataset is expressed as the temporal coherence in the rest recorded BOLD time-series. This inter-voxel connectivity data is then clustered, resulting in individual clustermaps. At the group level, the consistency across the individual clustermaps is clustered and this determines the group RSNs. As a result, the RSNs directly reflect groups of voxels that consistently showed a high level of functional connectivity across the group of subjects. A group of 26 subjects was scanned on a 3 Tesla scanner with a fast fMRI protocol. Analysis of the acquired resting-state data was done with the two-stage Ncut group clustering approach and the resulting group RSNs are discussed on their functional relevance, overlap and differences with previous reported resting-state studies.

## Results

**Figure 1:**
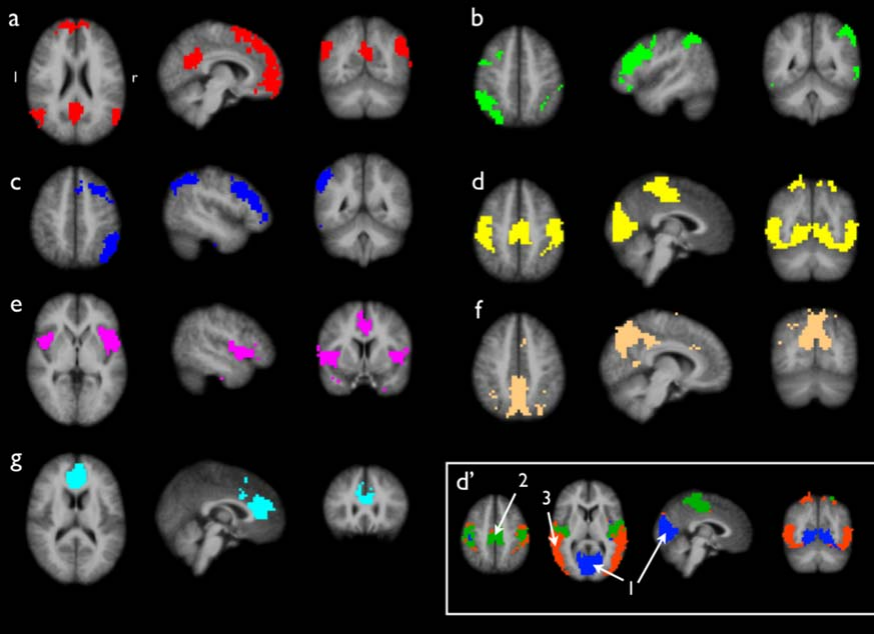
Group clustered resting-state networks. Group clustering of 3 Tesla resting-state fMRI data of a group of 26 subjects revealed 7 resting-state networks (RSNs). 1a shows a functional connected network consisting of the posterior cingulate/precuneus, medial frontal regions and bilateral parietal/temporal regions, a RSN known as the default mode network. 1b and 1c show lateralized parietal-frontal networks, networks that are often reported in attention and memory processing. 1d shows a joint network of both sensorimotor and visual networks. Iteratively clustering partitioned this cluster in 3 sub-clusters, shown in clustermap d'. The results showed separate clusters for primary visual regions (cluster *d*'*-1*), primary sensorimotor regions (cluster *d*'*-2*) and extra-striate visual regions (cluster *d*'*-3*). 1e shows a network of bilateral insular regions and posterior cingulate cortex. 1f and 1g represent singular clusters consisting of, respectively, a posterior part of Brodmann Area 7 and an anterior part of the cingulate cortex. The clustered networks show resemblance with previous reported RSNs.

Spatial maps of the 7 clusters are shown in Figure 1 and can be described as follows. Cluster *a* (Figure 1a) shows a network of posterior cingulate/precuneus region (Brodmann Area (BA) 23/31), middle temporal gyrus (BA 39), inferior temporal gyrus (BA 21), supramarginal gyrus (BA 40) and frontal regions, including both superior frontal gyrus (BA 8) and medial frontal gyrus (BA 11). Clusters *b* and *c* (Figure 1b and 1c) show highly lateralized parietal-frontal networks in the left and right hemisphere, involving superior parietal lobule, inferior parietal lobule, supramarginal gyrus (BA 7/40), middle frontal gyrus and superior frontal gyrus (BA 8/9). Cluster *d* (Figure 1d) shows the largest found network, consisting of postcentral gyrus (BA 3/1/2), precentral gyrus (BA 4), cingulate gyrus (BA 24) and lateral, medial and superior occipital gyrus and peristriate region (BA 17/18/19). Cluster *e* (Figure 1e) involves bilateral insular and superior temporal cortex (BA 22) and a part of the cingulate gyrus (BA 24). Cluster *f* (Figure 1f) involves a singular region consisting of a posterior part of BA 7. Cluster *g* (Figure 1g) involves a singular region covering a medial part of the medial frontal gyrus (BA 9) and an anterior part of the cingulate gyrus (BA 32).

**Figure 2:**
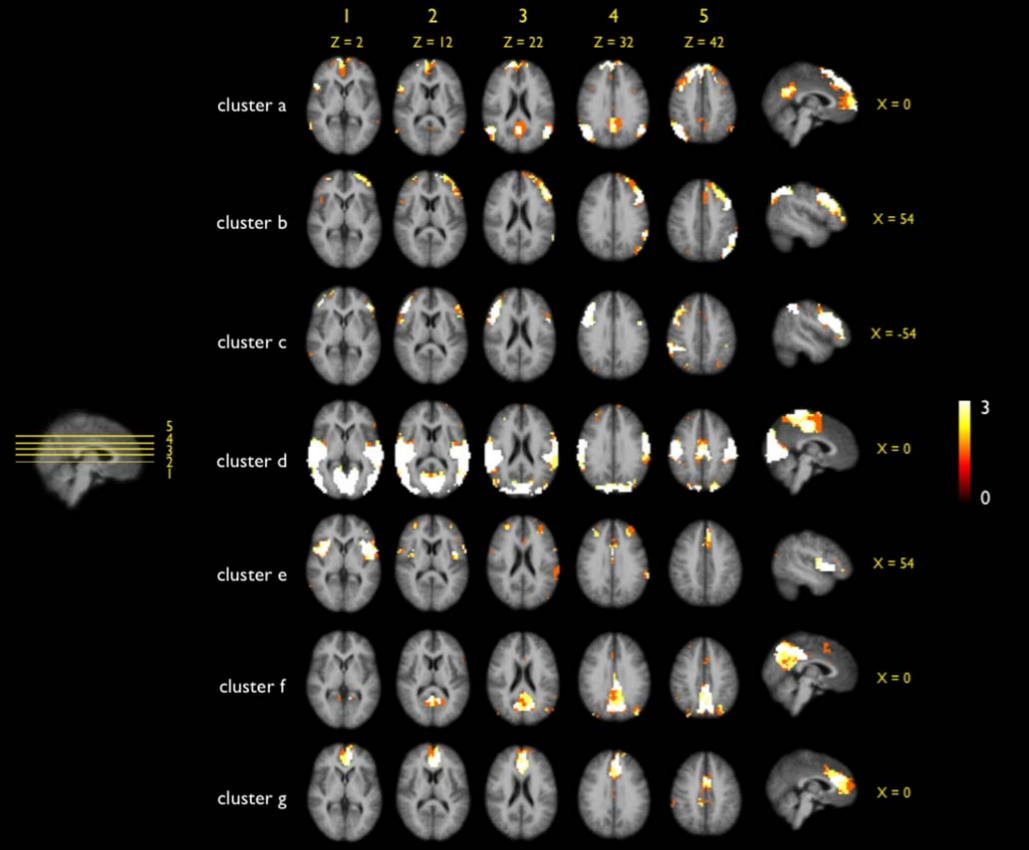
Overlap of multiple group clusterings of different sets of individual clustermaps, varying on the individual cut-off threshold. At the individual clustering stage, the constructed individual connectivity graph was threshold with the set individual graph cut-off threshold *rc* before clustering. To examine the effect of *rc* on the final group clustering, the individual clustering procedure was repeated with 3 settings of *rc*, being 0.3, 0.4 and 0.5. The overclustering parameter was kept fixed on 20. Group clustering (graph complexity threshold set to 9; number of RSNs set to 7) was repeated with the 3 sets of individual clustering results, resulting in 3 group clusterings. For all of the 7 group clusters the overlap of these clustering solutions was computed. Figure clearly shows large overlap for all of the 7 group clusters, indicating that the setting of *rc* did not affect the final group clustering. However, when *rc* was increased to 0.6 and up, more and more paths were removed from the individual graph. This clearly affected the individual clustering and the group clustering, changing the spatially layout of the clusters (data not shown).

**Figure 3:**
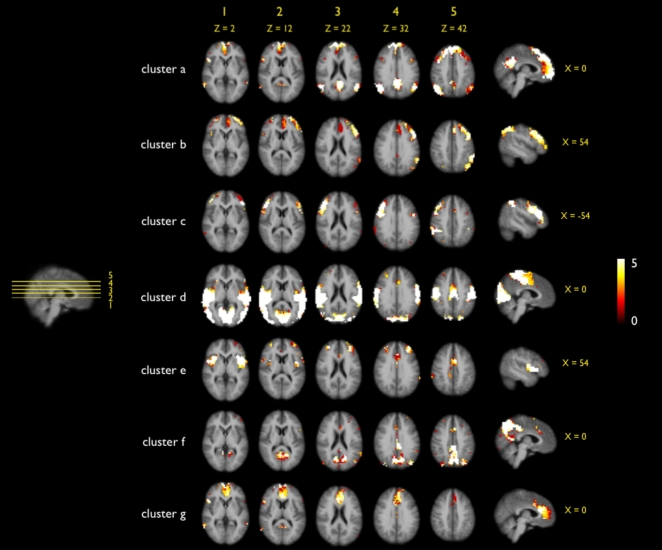
Overlap of multiple group clusterings of different sets of individual clustermaps, varying on the level of individual overclustering. To test the assumed minor effect of overclustering at the individual level on the group clustering results, the individual clustering (Stage A) was repeated with varying overclustering settings (i.e., the number of clusters) and analyzed at the group level (Stage B). For each individual dataset, the individual clustering (stage A) was repeated with varying number of clusters (assumed to result in overclustering), ranging from 15 to 35 (with steps of 5). This resulted in 5 clustermaps per individual dataset. Next, the group clustering (stage B) was repeated with the 5 sets of individual clustermaps (graph complexity threshold set to 9; number of RSNs set to 7). For each of the 7 resulting group clusters, the 5 cluster solutions were summated, creating an overlap map (cluster *a*–*g*) with voxel values ranging up to a maximum of 5. The maximum of 5 indicated an overlap off all 5 cluster solutions. Figure shows highly similar cluster results over the 5 different group clusterings, as suggested by the large overlap for each of the 7 clusters. As expected, the results demonstrated that the overclustering at the individual level did not change the nature of the group clustering.

Cluster *d* (Figure 1d) overlapped several brain regions. Iteratively clustering the voxels in cluster *d* with the Ncut group clustering approach revealed 3 sub-clusters within this cluster. The results of this sub-clustering are shown in Figure 1 (Figure 1-d', lower right corner). Cluster *d*'*-1* represents a sub-cluster consisting of striate and parastriate cortex (BA 17/18) (Figure 1d'-1). Cluster *d*'*-2* shows a sub-cluster consisting of postcentral gyrus (BA 3/1/2), precentral gyrus (BA 4) and cingulate gyrus (BA 24) (Figure 1d'-2). Cluster *d*'*-3* represents a sub-cluster consisting of lateral and superior occipital gyrus (BA 19) (Figure 1d'-3).

## Discussion

We report on a group clustering method to select resting-state networks at a group level. Voxels with coherent resting-state time-series were grouped and disconnected from voxels showing a different time pattern, resulting in individual clustermaps. At the group level, the consistency across the individual clustermaps was computed and clustered, defining the group resting-state networks (RSNs). Normalized cut group clustering of 3 Tesla resting-state fMRI data of 26 subjects revealed 7 independent functional connected resting-state networks.

Group clustering resulted in resting-state networks (RSNs) of known functional relevance that show resemblance with recently reported resting-state group ICA results. Six out of seven of the found clusters show resemblance with the group ICA results of Beckmann et al. [17], De Luca et al. [2] and Damoiseaux et al. [8], being cluster *a*, *b*, *c*, *d*, *e* and cluster *f*. Cluster *a* (Figure 1a) represents a network of regions that is often referred to as the default mode network [3], [5]. The default mode network is consistently found in resting-state fMRI studies [3], [21] and resting-state group ICA studies [2], [8], [17]. The default mode network is suggested to play an important role in core processes of human cognition [3], [4], [22]. Cluster *b* and *c* reflect lateralized parietal-frontal networks, which are often reported in attention and memory processing. Both networks are consistently found in resting-state studies [2], [8], [17]. Cluster *d* represents a combined network of regions involved in motor and visual processing. Cluster *e* shows a network consisting of bilateral insular regions and cingulate gyrus. This network is suggested to play an important role in the control of goal-directed behavior [23] and salience processing [24] and is often reported in RSN studies [2], [8], [17]. Two clusters, clusters *f* and *g* (Figure 1f and 1g), consist of singular regions, which suggest that these clusters show an isolated pattern of neuronal activity during rest. These results support the idea of complex functional connected RSNs underlying the architecture of the resting brain [25].

Interestingly, the group clustermaps also show important differences with previous reported resting-state studies. Previous studies have described up to 10 different RSNs [8], were in this study 7 RSNs were found. Most studies [2], [8], [17], [19] report the motor and visual regions to belong to two separate RSNs, while in this study they were grouped together in a single RSN (cluster *d*, Figure 1d). In addition, primary visual and extra-striate visual regions have also been reported to form separated RSNs [17], [26]. Clustering motor and visual regions in a single RSN suggests that other RSNs are more differentiated in their level of functional connectivity than motor and visual regions and could imply that these regions are interconnected to quite some extent. This marks an important differentiation between the results of this study and previous studies [2], [17], [19], [26] and this difference is particular noteworthy as a number of studies have especially foccused on the motor and visual system during rest and reported two separate networks [1], [6], [10], [11]. This suggested the existence of meaningful sub-RSNs within cluster *d.* The voxels in cluster *d* were therefore iteratively clustered using the Ncut clustering approach, partitioning cluster *d* in 3 sub-RSNs (see method section). This exploratory procedure resulted in 3 sub-clusters which are shown in Figure 1d' (lower-right part of Figure 1). Sub-clustering indeed resulted in two separate RSNs for primary visual regions (Figure 1d'-1) and primary sensorimotor regions (Figure 1d'-2), similar to previous group ICA [2], [8], [17] and clustering studies [19]. Furthermore, the extra-striate regions (Figure 1d'-3) were clustered as a separate RSN, similar as reported in Damoiseaux et al. [8] and Beckmann et al. [17], although clustermap *d*'*-3* overlaps somewhat larger regions. These results suggest a valuable role for iteratively clustering in the Ncut group procedure and future studies are aimed to further explore such a multilevel Ncut clustering approach.

A number of other differences between the results of this study and previous studies are of interest. First, the singleton cluster *f* (Figure 1f) was not found to be this prominent in other studies. Second, although most of the clustered RSNs represent regions of known functional relevance, cluster *g* (Figure 1g) does not directly correspond to a known functional network. Third, our results only partially overlap with the group results of Salvador et al. [25], who reports on a hierarchal graph clustering approach of resting-state fMRI. In their study, six main clusters were found, of which only the motor and visual clusters show some overlap with our combined motor/visual network (cluster *d*, Figure 1d). The differences in clustering results may arise from the used voxel-wise approach in our study, in contrast to the averaged regional approach used by Salvador et al. In addition, in the study of Salvador et al. the group connectivity graph was constructed by averaging the individual regional-connectivity graphs, while in this study the group graph reflected the cluster-consistency over the individual clustering results.

Overall, despite differences between studies, we take the similarities between the clustered group networks and the previous reported group ICA components as a demonstration of the robust formation of functional networks when the brain is at rest. In this study, resting-state data was acquired in a different setting (on a 3 Tesla scanner and with a six times faster fMRI protocol) and analyzed with a novel group clustering approach, but this still resulted in the selection of known RSNs. The RSN similarities marks the potential of our normalized cut group clustering method in correctly detecting functionally connected RSNs in the resting brain.

**Figure 4:**
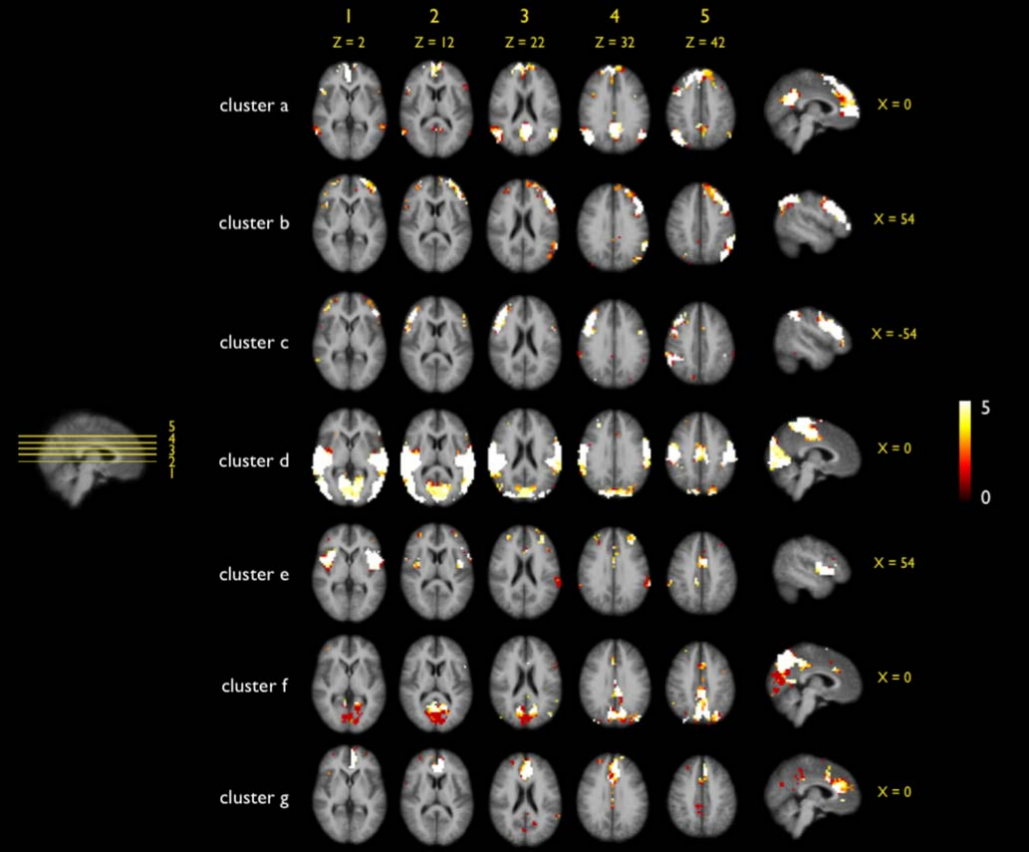
Overlap of multiple group clusterings with varying group graph complexity cut-off thresholds. To verify that the optimization procedure considering the selection of the group graph complexity cut-off threshold resulted in a stable clustering, the group clustering was repeated with multiple settings of the graph complexity cut-off threshold. The group clustering stage (stage B) was repeated (using the individual clustermaps consisting of 20 clusters) with different settings for the group cut-off threshold varying around the found optimum of 9 (ranging from 7 to 11). The number of clusters was set to the found optimum of 7 (see main text). Next, for each of the 7 clusters, the 5 group cluster solutions were summated, creating an overlap map with voxels ranging up to a maximum of 5. The maximum of 5 indicated the overlap of all 5 clustering solutions. Figure shows large overlap between the 5 group clusterings, for all of the 7 clusters. This large overlap demonstrates that varying the cut-off threshold around the found optimum only minor influenced the group results, indicating that the automatic parameter setting procedure resulted in a stable clustering solution.

The consistency of the proposed Ncut clustering group approach was studied by examining the results of multiple clusterings with different parameter settings (Figure 2, 3 and 4). First, the cut-off threshold of the individual connectivity graph was varied for three settings (being 0.3, 0.4 and 0.5). This resulted in three different sets of individual clustering results which were clustered at the group level (Stage B). The group clustering showed to be only minor influenced by the individual cut-off threshold, as all 7 clusters of the 3 sets showed high overlap (Figure 2). However, when the individual cut-off threshold was increased to 0.6 (and up) more and more paths were removed from the individual graphs and this did have an effect on the group results, showing less consistent group RSNs. Therefore the individual cut-off threshold was set to 0.4 in the main analysis. Second, the influence of the level of overclustering at the individual clustering stage (Stage A) on the final group clustering was examined by repeating the clustering with 5 settings of overclustering, ranging from 15 to 45. This resulted in 5 sets of group clusterings, all showing large overlap for all of the 7 clusters (Figure 3), indicating that changing the level of individual overclustering did not change the nature of the group clustering results. As a third test, the influence of the group-graph complexity parameter on the group clustering was examined. The group graph was clustered with 5 settings of the group cut-off threshold around the found optimum of 9. The 5 cluster solutions showed large similarity for all of the 7 group clusters (Figure 4). This overlap suggests only a minor influence of the graph complexity threshold on the group clustering. Taken together, these results show that the used Ncut group clustering approach yield consistent results for clustering with different parameter settings. This indicates that our group clustering approach is robust for different settings of the cluster parameters. In addition, the overlap suggest that the clustered RSNs can be found consistently in a group of subjects, increasing the confidence in the found RSNs.

A number of model-free methods have been successfully introduced for the group wise selection of RSNs from resting-state fMRI. ICA based methods are perhaps the most commonly used [17], [18] and have been reported to show large consistency [27], [28]. ICA methods search for a mixture of sources underlying the observed signal, with the assumption that the sources are statistically independent. An advantage of these methods is that they work at the voxel level and that the temporal signal of the independent components can be easily further examined and compared between groups. However, the interpretation of the ICA results may involve human input in selecting the anatomical meaningful networks from the total collection of components and this can be a complex task. New analysis methods are suggested to calculate the consistency across ICA solutions to provide additional information for a better interpretation of the results [28]. Clustering methods have also been successfully used to investigate RSNs [15], [19]. Salvador et al. [19] reported on hierarchical graph clustering of an averaged group connectivity matrix, clustering brain regions in six main groups. Advantages of this clustering method are the simplified selection of RSNs by controlling for the hierarchical levels of clustering and the straightforward interpretation of the results. However, most clustering approaches have clustered over brain regions, using a parcellation of the cortex in a number of fixed regions, making the spatial resolution of these methods limited to a regional scale. Clustering at the group level forms the core of our group clustering approach. Functional connectivity is represented on the voxel scale, enabling the examination of RSNs in detail, similar to group ICA methods [17], [18]. A strong asset is the data-driven computation of the number of RSNs, avoiding human input in defining the number of RSNs. Clustering purely implies the grouping of voxels that consistently show correlated time-series across a group of subjects making the results straightforward to interpret. However, in contrast to group ICA methods, the temporal signals of the RSNs are not directly available for further processing, but this requires some level of post analysis like overlapping the clusters on the functional time-series and calculating the level of (partial) correlation between the RSN regions. Graph clustering methods using minima criteria, among which hierarchal clustering and k-means clustering, tend to be sensitive to outliers in the dataset [29]. Minima based clustering methods are generally very effective when clustering averaged time-series over regions, as noise over the time-series is averaged out. However, such clustering strategies are less effective in clustering voxels. The time-series of a single voxel could have a low signal to noise ratio and therefore easily show a rather distinct pattern from the rest of the dataset. As a result, the clustering of voxel-based data could result in grouping such an outlier or a small group of outliers as single clusters, ignoring the more global character of the data [15], [29]. To overcome this problem, in this study the normalized cut clustering of Shi and Malik [20] was used. The normalized cut criterion measures both the total similarity within groups as well as the dissimilarity between groups, effectively penalizing the formation of small clusters and thereby stimulating the clustering of more global RSNs.

The neurophysiological meaning of resting-state networks remains unclear. It has been proposed that synchronization of neuronal oscillation patterns within neuronal networks may contribute to the regulation of information flow [30] supporting selection, consolidation and combination of learned information [31], processes that are likely to be on-going during rest [32], [33]. Coherent oscillatory patterns during rest in RSNs may therefore be involved in the consolidation of past events and the preparation for future responses to stimuli [32], [33]. More specific, robust functional connections between the posterior and anterior cingulate cortex of the default mode network have been associated with integration of cognitive and emotional processing [3].

Investigations into the resting-state of the brain may give us more insight into the foundation of the brains architecture and its dysfunction in brain disorders. It has been recently suggested that resting-state patterns may be affected in Alzheimer's disease [34], [35], depression [36] and schizophrenia [37]–[42]. Our proposed group clustering method could contribute to this field of research. It allows for a direct comparison of the spatial distribution of RSNs between patients and healthy controls. Furthermore, the used graph representation of connected voxels can be used to examine the organization of the functionally connected resting-state brain [19], [43] and possible disruptions in network organization in patients [39].

The described two-stage Ncut group clustering approach requires to set a number of parameters by the user before clustering, being the individual cut-off threshold, the level of individual overclustering and the group graph complexity threshold. In this study, the influence of the chosen parameter settings was tested and found to have only a minor influence on the group clustering (Figure 2, 3 and 4). In addition, the number of group clusters, defining the total number of group RSNs, has to be defined. The described approach includes an exhaustive search procedure to find the group cluster parameters that resulted in an optimal clustering of the group data, by finding a clustering solution with a minimal normalized cut cost [20]. The examined influence of the individual and group parameters on the final group clustering and the described optimization procedure might assist the user to set the required parameters.

Some limitations to this study have to be considered. Like in all clustering based resting-state methods, all cortical voxels must be placed in one of the RSNs. However, it can be argued that only parts of the brain participate in RSNs. Indeed, two of the found clustermaps show a solitary group of voxels, which suggest that these voxels do not participate in a network, but rather show an isolated pattern of neuronal activity during rest. Stronger pre-selection criteria may address this issue by removing voxels that show little to no significant connections from clustering. An additional limitation of using a clustering approach is that each voxel is exclusively assigned to one cluster. This may be incorrect for regions that participate in multiple RSNs, for example subcortical regions with relay functions like the thalamus. In this study we focused exclusively on the cortical areas, but it is of interest to examine subcortical contributions to RSNs. A more general limitation considers the assumption of cross-correlation between time-series reflecting functional connectivity. Two voxels showing a high cross-correlation between their time-series could be mediated by a third voxels time-series and not because they are (directly) functionally connected. The use of partial correlation or partial coherence has been suggested to account for these third party influences [15], [19], [44], [45].

We introduced a two-stage normalized cut based group clustering method to investigate the formation of resting-state networks in the human brain at a group level. Inter-voxel functional connectivity was clustered into individual networks and consistency of these networks over the group of subjects determined the group RSNs. Group clustering of rest recorded 3 Tesla fMRI data of 26 subjects revealed resting-state networks of known functional relevance and included the often reported default mode network. Our results support the idea of the formation of spatially distinct RSNs during rest in the human brain.

## Materials and Methods

### Datasets and preprocessing

Data was acquired using a 3 Tesla Philips Achieva Medical scanner (Philips Medical Systems, Best, The Netherlands) at the University Medical Center Utrecht, The Netherlands. 26 right handed healthy subjects with no psychiatric history participated in the study (age mean/std : 25/7.7; gender: 14 male, 12 female). All participants gave written consent prior to taking part in the study as approved by the medical ethics committee for research in humans (METC) of the University Medical Center Utrecht, the Netherlands. During the resting experiment the scanner room was darkened and the subjects were instructed to relax with their eyes closed, without falling asleep. Resting-state blood oxygenation level dependent (BOLD) signals were recorded during a period of 8 minutes using a fast fMRI sequence (3D-PRESTO pulse sequence with parallel imaging [46], [47]. Acquisition parameters: TR 21.75 ms, effective TE 32.4 ms (using a shifted echo); flip-angle 9 degrees; 1000 timeframes; FOV 256×256 mm, voxelsize 4 mm isotropic, 32 slices covering whole brain; total acquisition time per volume 0.5 sec). The short volume acquisition time of 500 ms allowed the sampling of information in the frequency domain up to 1 Hz. This minimized the possible backfolding (aliasing) of higher frequencies, such as cardiac and respiratory oscillations into the lower resting-state frequencies of interest (0.01–0.1 Hz). Directly after the acquisition of the functional time-series an additional PRESTO scan with better anatomical contrast using an increased flip angle of 25 degrees (FA25) was acquired for coregistration purposes. A T1 weighted image was acquired for anatomical reference (3D FFE pulse sequence. Acquisition parameters: TR = 9.87 ms, TE = 4.6 ms; flip-angle 8 degrees; SENSE reduction 1.7 (left-right) and 1.4 (anterior-posterior); FOV 240×240 mm, voxelsize 0.75×0.75×0.8 mm, 180 slices).

All preprocessing steps were done with the SPM2 software package (http://www.fil.ion.ucl.ac.uk). The functional scans were corrected for small head movements by realigning all functional scans to the last functional scan. Realignment to the last functional scan ensured maximum spatial overlap with the FA25 scan at the start position of the registration, because the FA25 scan was acquired directly after the resting-state time-series. The functional time-series were coregistered to the FA25 image, by taking the last functional scan as a source. The T1 image was then coregistered to the FA25 image, providing spatial alignment between the functional time-series and the anatomical image. After realignment, the rest recorded functional time-series were bandpass filtered with a finite impulse response (FIR) bandpass filter with zero phase distortion (bandwidth 0.01–0.1 Hz) to eliminate low frequency noise (including slow scanner drifts) and influences of higher frequencies reflecting possible cardiac or respiratory oscillations [11]. Normalization parameters were estimated using the MNI 305 T1 brain [48] as a template and the T1 image as a source. All functional scans were then normalized to the standard space according to normalization parameters and resampled to a 4×4×4 mm resolution, enabling between subject comparisons. Cortex segmentation was done on the T1 image using the Freesurfer software package (http://surfer.nmr.mgh.harvard.edu/). Segmentation maps were normalized and resampled to a 4×4×4 mm resolution to spatially overlap the filtered time-series.

After preprocessing, the resting-state fMRI datasets were analyzed using the normalized cut group clustering method, consisting of two clustering stages (explained in detail below). At the individual level (Stage A, Individual clustering), voxels showing correlated BOLD activation patterns over time were clustered and defined an individual clustermap. Next, at the group level (Stage B, Group clustering) the consistency across the individual clustermaps was computed and clustered using the Ncut method, defining the group RSNs.

### Stage A: Individual clustering

**Figure 5:**
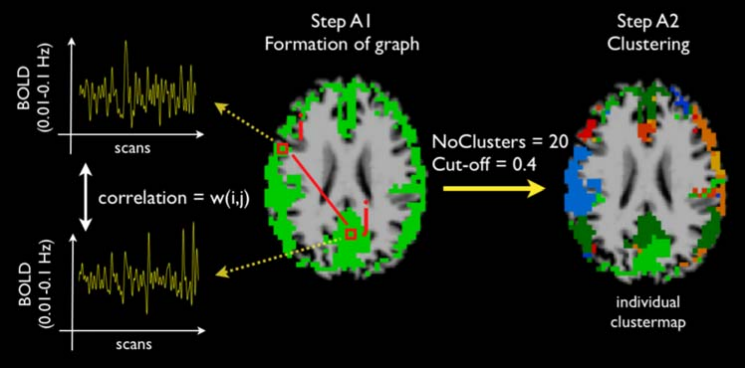
Individual clustering stage. *Step A1 Graph formation*. An individual graph was constructed, consisting of M cortical voxels and (M^2^ -M)/2 edges connecting all voxel pairs. The weight *w*(*i,j*) of edge *e*(*i,j*) connecting voxel *i* and voxel *j* was computed as the correlation between the filtered time-series of voxel *i* and voxel *j*, reflecting the level of functional connectivity between the two voxels. *Step A2 Clustering*. Prior to the clustering, a cut-off threshold of 0.4 was applied to reduce the complexity of the graph and lower the computational load, setting all weights to zero that did not reach this threshold. Normalized cut clustering was used to partition the graph in a fixed number of 20 networks, grouping voxels that showed a high level of functional connectivity into networks, resulting in an individual clustermap.

Clustering at the individual level (Stage A) involved the grouping of voxels that showed coherent BOLD fMRI time signals. The individual clustering stage consisted of two steps (Figure 5), the *Formation of an individual functional connectivity graph* (step A1) and the *Clustering* of this graph (step A2).

#### Step A1 Formation of an individual functional connectivity graph

Each fMRI dataset was represented as a fully connected undirected graph *G* = (*N,E*), with nodes N representing the voxels in the dataset (all gray matter voxels) and the weighted edges E connecting each voxel pair (Figure 5, step A1). This resulted in an individual graph of M nodes representing the M cortical voxels with a total of (*M* ^2^−*M*)/2 edges connecting each possible voxel pair. M varied between subjects from 8500 to 9500, depending on the individual cortical segmentation. The weights of the edges of the graph represented the level of functional connectivity between the voxels. The weight *w*(*i,j*) of edge *e*(*i,j*) connecting voxel *i* and voxel *j* in the graph was computed as the correlation between their filtered fMRI time-series. The individual functional connectivity graph was stored as a weighted connection matrix before clustering.

#### Step A2 Clustering

Next, the connectivity graph *G* was clustered in a number of sets consisting of groups of voxels that showed a high level of functional connectivity (Figure 5). To partition graph G in a number of subsets the normalized cut-cost clustering method according to Shi and Malik [20] was used. By using a *graph cut method* for clustering a graph is partitioned in a number of subsets by removing edges from the graph that connect the subsets, with the total cost of this operation defined as the sum of all the weights of the edges that have to be removed. The normalized cut value normalizes this cut cost by the fraction of all paths in that subset (for a more detailed description see supporting information text S1). A direct advantage of the Ncut method is that, due to the normalization factor, the clustering is less sensitive to grouping outliers as individual subsets. The optimal partitioning of graph G is one that minimizes the Ncut cost and can be found by solving a generalized eigenvalue system representation of G [20]. Clustering was done with the public available MALTAB implemented Ncutclustering_7 toolbox of Shi (http://www.cis.upenn.edu/jshi/software).

To reduce the number of connecting edges in the connectivity graph and therefore reduce the graph complexity, a cut-off threshold *rc* [15] of 0.4 was applied, setting the weights of the edges that did not reach this threshold to zero. The effect of this individual cut-off threshold on the group clustering was examined by repeating the individual clustering stage with 3 different settings of *rc,* being 0.3, 0.4 and 0.5, resulting in 3 clustermaps for each individual dataset. The group clustering stage (Stage B, see below) was then repeated with the 3 different sets of 26 individual clustermaps, resulting in 3 group clusterings. Varying the individual cut-off threshold *rc* around 0.4 did not change the nature of the group clustering, as indicated by the overlap of the resulting group clusters (Figure 2). Therefore, *rc* was set to 0.4.

Clustering required a preset number of clusters to partition in. At start the number of RSNs was unknown. Setting the number of clusters lower than the true number of RSNs would result in an underclustering of the data, erroneously combining distinct RSNs. Setting a high number of clusters would probably result in an overclustering of the data, forcing networks to be divided in multiple subsets. However, if overclustering of the data would force a true RSN to be split in two or more subsets, the assignment of the voxels to one of the subsets would be random, as no correct splitting would be possible on basis of the data itself. Therefore, the devision into subsets would not change the shape or outline of the RSNs and would not affect the nature of the group clustering. To verify this assumed minor effect of individual overclustering on the group clustering, the individual clustering stage was repeated with the (overclustering) number of clusters ranging from 15 up to 35 (with steps of 5), resulting in a total of 5 clustermaps per individual dataset. Subsequently, the group clustering step (Stage B, see below) was repeated 5 times (using fixed group cluster parameters), with the 5 different sets of 26 individual clustermaps. As predicted, the 5 group clustermaps demonstrated large overlap, indicating that overclustering at the individual level did not change the nature of the group results (Figure 3).

To avoid incorrect underclustering of the data, the number of clusters was set to 20, double the number of 6 to 10 networks reported in previous fMRI resting-state studies. This was expected to result in (harmless) overclustering of the data. The individual graph was partitioned in 20 clusters using the Ncut clustering algorithm. Labeling the cortical voxels with their cluster number (ranging from 1 to 20) resulted in an individual clustermap. Small gaps in the individual clustermap were filled by using a majority voting algorithm with a minimum of 5 neighbours.

### Stage B: Group clustering

**Figure 6:**
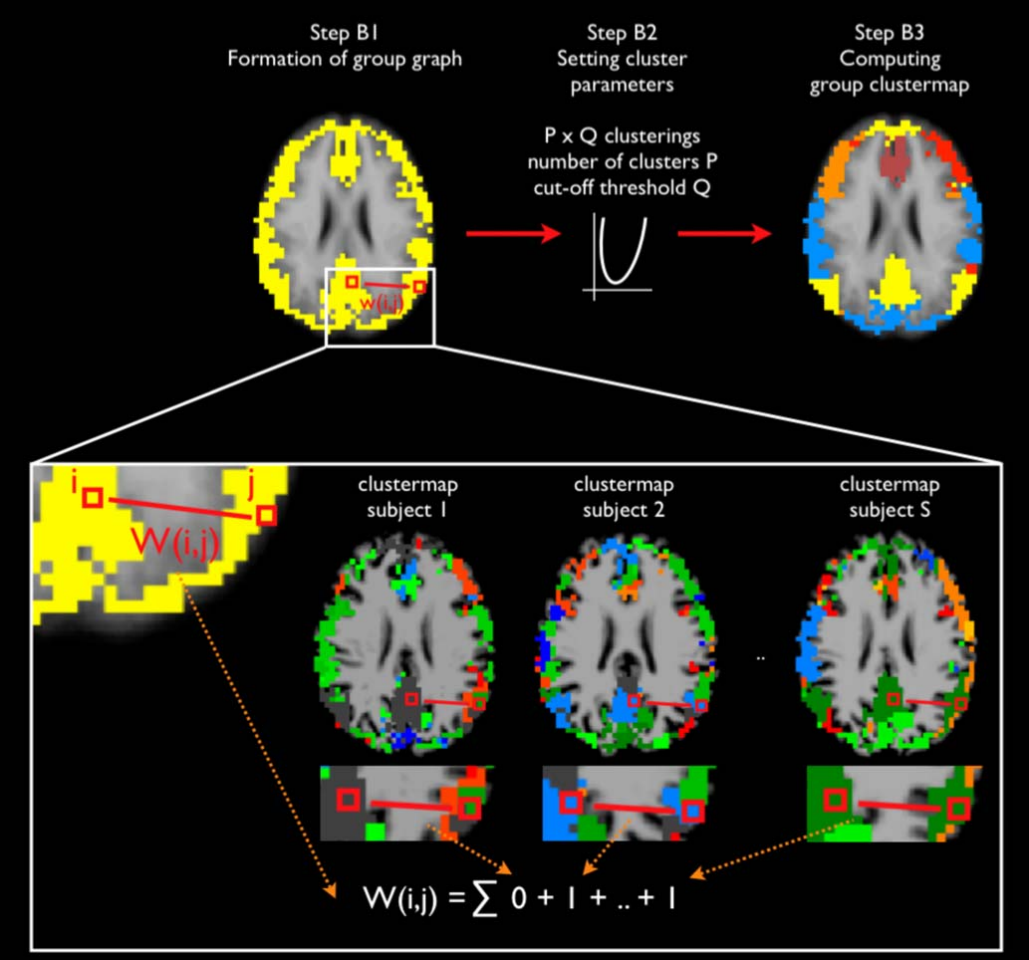
Group clustering stage. *Step B1 Formation of a group graph*. A group graph was constructed, consisting of the cortical voxels that resulted from the group averaged cortical segmentation map and edges connecting all possible voxel pairs. The weight *W*(*i,j*) of the edge connecting voxel *i* and voxel *j* reflected the cluster consistency across the group of subjects and was computed as follows. For each individual clustermap, the individual cluster-similarity between voxel *i* and *j* was defined as 1 if in the individual clustermap voxel *i* and voxel *j* were grouped in the same cluster and 0 otherwise. Figure box shows the clustermaps of subject 1 and 2 and the last subject (subject S). In subject 1 the voxels *i* and *j* were not clustered in the same cluster, hence the cluster-similarity between voxel *i* and voxel *j* was set to 0. In contrast, in subject 2 and in subject S voxel *i* and *j* were clustered in the same cluster and therefore the cluster-similarity values between these voxels in these subjects were set to 1. At the group level, *W*(*i,j*) was computed as the summation of the cluster-similarities between voxel *i* and voxel *j* over the group of S subjects. *Step B2 Setting cluster parameters*. The group graph was clustered with increasing number of clusters P and increasing graph complexity cut-off thresholds Q. An optimal fit was computed as the clustering fit with the first minimum normalized cut cost value in descending direction of the number of P clusters, to maximize the number of meaningful clustered RSNs. *Step B3 Computing group clustermap.* The cortical voxels were labeled according to the optimal clustering fit, resulting in the group clustermap. The group clustermap represents networks of voxels that were consistently clustered into the same resting-state network across the group of subjects.

In the group clustering stage the consistency across the 26 individual clustermaps was computed and clustered (Stage B, Figure 6). The resulting group clustermap expressed networks that could be consistently found across the group of subjects. Group clustering consisted of 3 steps, *Formation of the group graph* that reflected the RSN consistency across the group of subjects (step B1), *Setting cluster parameters* (step B2) and *Computing the group clustermap* (step B3).

#### Step B1 Formation of the group graph

Overlap of the normalized individual cortical segmentation maps resulted in a group cortical segmentation of 9014 gray matter voxels. From the 9014 cortical voxels a fully connected undirected group graph *Ggrp* was constructed with (9014^2^−9014)/2 edges connecting all voxel pairs (Figure 6, step B1). The weights of the edges were set to express the consistency of the cluster-similarity across the individual clustermaps and were computed as follows. In each individual clustermap (the outcome of the individual clustering stage), the cluster-similarity between two voxels was set to 1 if the two voxels were grouped in the same cluster and 0 otherwise. Then at the group level, the weight of the edge connecting those two voxels in the group graph was computed as the total summation of the cluster-similarity values across the individual clustermaps. More formally, weight *W*(*i,j*) of edge *E*(*i,j*) between voxel *i* and *j* in *Ggrp*, was defined as
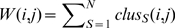
with N the number of subjects and *clus_S_(i,j)* being 1 if voxel *i* and *j* were in the same cluster in subject S and 0 otherwise. This definition of the weights of the group graph expressed between subject cluster-similarity in such a way that high weight values reflected strong subject overlap. A high value of *W*(*i,j*) would indicate that in a large proportion of the group of 26 subjects the two voxels *i* and *j* were clustered into the same cluster and therefore should probably belong to the same group RSN.

#### Step B2 Setting cluster parameters

At start of the group clustering stage the number of clusters (i.e. the number of RSNs) to partition *Ggrp* in was unknown. Furthermore, a graph complexity cut-off threshold was needed to be set to decrease the size of *Ggrp* in preparation for the clustering to reduce the computational load, setting the weights of the edges that did not reach the complexity cut-off threshold to zero. At start, the number of group clusters and the cut-off threshold were unknown and as a result could not be set correctly by the user. An optimal partitioning of *Ggrp* with respect to these 2 parameters was computed with the following procedure. First, *Ggrp* was clustered repetitively with different cut-off thresholds and with different values for the number of clusters (i.e. RSNs). For each of these clusterings the Ncut method was used and the Ncut cost to partition *Ggrp* was computed. This resulted in a *Ncut cost landscape* of size P×Q, with P the range of used numbers of clusters and Q the range of used graph complexity thresholds. The examined number of group clusters varied between 2 and 50 clusters and the graph complexity threshold varied between 5 and 25, resulting in a 49×21 Ncut cost landscape, containing 1029 group clustering solutions. Second, from this Ncut landscape the optimal clustering solution was selected. The Ncut cost of a clustering is defined as the summation of the weights of the edges that have to be removed to divide the group in multiple sets and directly reflects the quality of the clustering [20]. Therefore, the optimal partitioning of *Ggrp* in an optimal number of clusters, was selected from the total collection of cluster solutions as a solution with a minimum Ncut cost. This minimum was selected by traveling through the Ncut cost landscape in descending direction of the number of clusters starting with 50 (i.e. from 50 to 2) -partitioning the data in as much meaningful networks as possible- and in ascending direction of the cut-off threshold (i.e up from 2 to 25), keeping the information in *Ggrp* as high as possible. This procedure resulted in an optimal partitioning of *Ggrp* in 7 clusters with a group cut-off threshold of 9.

#### Step B3 Computing the group clustermap

The cluster labels resulting from the computed optimal clustering of *Ggrp* were assigned to the cortical voxels (Figure 6, step B3). This resulted in a group clustermap of 7 RSNs.

To confirm that the optimization procedure resulted in a stable clustering fit of *Ggrp*, the overlap of the clustering solutions with a cut-off threshold varying around the found optimum of 9 was calculated. A range of −2 to 2 was chosen (i.e. 5 cut-off thresholds ranging from 7 to 11). To test the specific effect of the cut-off threshold on the group clustering the number of clusters was kept fixed for all 5 group clusterings to the found optimum of 7. The 5 computed group clustermaps showed large overlap for all of the 7 clusters, indicating that the optimization procedure resulted in a stable clustering (Figure 4).

### Additional analysis, sub-clustering of cluster d

The largest clustered network (cluster *d*, Figure 1) consisted of both sensorimotor and visual regions, combining these regions in a single RSN. To examine whether this cluster consisted of multiple sub-clusters, an iterative cluster procedure was added, sub-clustering the voxels in cluster *d* (Figure 1d). This iterative clustering stage followed the exact procedure of the normalized group clustering approach, with both the individual clustering stage (Stage A) and group clustering stage (Stage B). First, individual connectivity graphs were formed out of the selected voxels and clustered. For the individual clustering a cut-off *rc* of 0.4 was used and the level of overclustering was set to 10 clusters, being over twice the expected number of clusters, similar to the procedure followed in the main analysis (Stage A: Individual clustering, step A2). The individual clusterresults were combined at the group clustering stage, forming a new group graph, consisting of 4186 voxels (Stage B: Group clustering). This graph was then clustered with the graph-complexity threshold set to a value of 9 (similar to the main analysis) and the number of group clusters set to 3.

## Supporting Information

Text S1 (0.05 MB DOC)
