# Supplementary material for: Normalized Cut Group Clustering of Resting-State fMRI Data
Source: PLoS One. 2008 Apr 23;3(4):e2001. doi: 10.1371/journal.pone.0002001 (PMC2291558; doi:10.1371/journal.pone.0002001)
Supplement: Text S1 — (0.05 MB DOC) [file pone.0002001.s001.doc]

## Supporting Information text S1

*Normalized cut cost*

A graph is a mathematical object defined by G = (N,E), consisting of a collection of nodes N, a collection of edges E connecting the nodes, with *w*(*u,v*) representing the weighted dependency between node *u* and *v* of edge *e*(*u,v*)*.* G can be cut into two disjoint subsets A and B with , by removing the connecting edges between the nodes in A and the nodes in B, with a total cut cost of

(1)

An optimized partitioning of G is defined as a partioning that minimizes the total cut cost (1) [1]. However, partitioning G in *k* subsets by minimizing (1) may result in grouping small sets of isolated nodes as subsets, as (1) increases with the number of edges across A and B [1].

Shi and Malik [1] introduced the *normalized cut cost* (*Ncut*), in which the cut cost of dividing G in subset A and B is defined as a fraction of the total weights of all the nodes in G of *cut*(*A,B*):

(2)

with *assoc*(*A,N*) expressing the total collection of weights from nodes in subset A with all other nodes in G:

(3)

From (2) it follows that grouping a small number of nodes as a subset will no longer give a low cut cost, as this will certainly be a large fraction of all the edges of that small subset to the other nodes in G. In a similar way, G can be grouped in *k* subgroups with the Ncut cost defined as:

(4)

*Optimal partitioning*

For a connected graph G = (N,E) to be divided in two subsets A and B, let *x* be a indicator vector of size N x 1, with $x_{i}=1$ if node $x_{i}$ is in A and -1 otherwise. Now, let

$d(i)=\sum w(i,j)$ be the total summation of all connections from node *i* to all other nodes in N of G,

D a matrix of size N x N with *d* on the diagonal,

W a connection matrix of graph G, with $W(i,j)=w_{\text{ij}}$ and

*b* defined as.

Now, Shi and Malik [1] showed that an optimal partition of G with respect to minimizing the Ncut cost value can be found by solving the generalized eigenvalue system

(5)

with and 1 a vector of size N x 1. Shi and Malik showed that the second to smallest eigenvector of this generalized eigenvalue system (5) reflects the real value solution of dividing G in subsets A and B with respect to a minimalization of the Ncut cost. Similar, it can be shown that solving (5) will result in an optimal partitioning of G in *k* subsets.

*References*

1. Shi J, Malik J (2000) Normalized cuts and image segmentation. IEEE Transactions on pattern analysis and machine intelligence 22: 17.
